# Editorial: Construction of critical nutrient concentration model for precision agriculture

**DOI:** 10.3389/fpls.2023.1286466

**Published:** 2023-09-27

**Authors:** Zunfu Lv, Syed Tahir Ata-Ul-Karim, Guoquan Lu

**Affiliations:** ^1^ The Key Laboratory for Quality Improvement of Agricultural Products of Zhejiang Province, College of Advanced Agricultural Sciences, Zhejiang A&F University, Hangzhou, China; ^2^ Graduate School of Agricultural and Life Sciences, The University of Tokyo, Tokyo, Japan

**Keywords:** critical n concentration, nutrition index, sweetpotato, wheat, critical dilution curves, critical K concentration

Nutrients, such as nitrogen, phosphorus and potassium, have an important influence on plant growth. Plant growth models such as fertilizer demand diagnostic models play a vital role in plant cultivation and production. The fertilizer demand models are often constructed based on critical dilution curves to achieve precise agriculture and avoid nutrient deficiencies or surpluses in plants, plant cultivation, and management. In recent years, the critical N concentration and dilution models have been established for multiple crops (Lv et al., 2020). However, critical dilution curves are subject to dynamic interactions with nutrition elements, genotype, environment and techniques, which can lead to slight differences between different sites. Analyzing the interaction relationship between nutrient elements based on the critical nutrient curve can clarify the interaction mechanism between elements (Lemaire et al., 2019). In addition, the combination of remote sensing technology and critical nutrient concentration can achieve rapid diagnosis of crop nutrition. Therefore, the goal of this Research Topic is to construct a critical nitrogen, phosphorus, and potassium dilution curve for various crops, clarify the interaction relationship between elements, construct nutrient diagnostic models and nutrition demand models, and achieve a rapid diagnosis method for crop nutrition based on remote sensing. This Research Topic contains five articles, each of which offered new insights on critical dilution curves and precision agriculture.

In recent years, researchers have primarily focused on studying critical nitrogen and phosphorus concentration models, leaving the construction of critical potassium concentration models relatively understudied. However, He et al. conducted a study specifically targeting the construction of a critical potassium concentration model for sweetpotato. Their research aimed to determine the nutritional status of sweetpotato crops by analyzing changes in the critical K dilution curve (CKDC) under varying nitrogen levels. Additionally, they sought to gain a better understanding of the nitrogen-potassium interaction mechanism in sweetpotato plants. By measuring and analyzing potassium concentrations in sweetpotato plants grown under different nitrogen levels, the researchers successfully constructed the CKDC model. This model provided valuable insights into the critical potassium concentration at different growth stages of sweetpotato. Ultimately, this study contributes to the existing knowledge on critical nutrient concentrations in plants, specifically highlighting the significance of potassium in sweetpotato cultivation. The findings have practical implications for farmers and researchers, as they can utilize the critical potassium concentration model to assess crop nutritional status and optimize fertilizer application for improved growth and yield.

According to Ata-Ul-Karim et al. (2016), nitrogen (N) fertilizer is crucial for enhancing the yield and quality of major cereals. It is essential to understand the relationship between crop growth and N demand during the growth period to optimize N scheduling decisions and improve N use efficiency. Fertilizer demand models are often developed based on critical dilution curves to achieve precise fertilization practices. In their study, Li et al. aimed to establish the relationship between accumulated nitrogen deficit and agronomic N use efficiency for winter wheat. The results revealed significant variations in accumulated N deficits across sites, growth stages, and cultivars under different N rates. Accumulated nitrogen deficit effectively reflected the internal N status of the plants, indicating its potential to quantify in-season crop N status for winter wheat. Furthermore, accumulated nitrogen deficit showed a positive correlation with N use efficiency. The validation results demonstrated the accurate prediction of in-season N use efficiency using the newly developed models. These findings can contribute to improving in-season N use efficiency by refining N scheduling decisions in intensive wheat cropping systems in China.

The nitrogen nutrition index (NNI) serves as a crucial indicator for diagnosing crop nitrogen nutrition. It quantitatively reflects the abundance or deficiency of nitrogen in crops, enabling accurate diagnosis and providing guidance for fertilization decisions. Remote sensing technology allows for quick estimation of NNI. However, the traditional calculation of NNI relies on expensive and time-consuming field sampling for nitrogen content and biomass data, resulting in delayed results and limited applicability in actual crop production (Liu et al., 2019). In recent years, the emergence of airborne hyperspectral technology has revolutionized precision agriculture by enabling the acquisition of physical and chemical information on rice through UAV hyperspectral remote sensing (Chanseok et al., 2011). This technology analyzes spectral data to obtain crop growth information, offering advantages such as speed, accuracy, and minimal loss of information (Zhang et al., 2021). In the research conducted by Yu et al., unmanned aerial remote sensing technology was utilized to acquire rice canopy spectra for estimating the nitrogen nutrition index (NNI) of rice crops. The authors determined the NNI by creating a critical nitrogen concentration curve specific to rice. They further processed the canopy spectra through log, difference, and log-difference transformations, based on the critical nitrogen concentration spectra. After extracting features using a self-encoder, the Extreme Learning Machine and Bagging Extreme Learning Machine algorithms were employed for modeling purposes. This research introduces an innovative and viable methodology for predicting the spectral NNI of rice, offering a promising approach to estimate the nitrogen nutrition index of rice crops.

Improved monitoring of nitrogen content in leaves is crucial for calculating the Nitrogen Nutrition Index (NNI) for accurate nitrogen nutrition diagnosis. In the study conducted by Qin et al., they developed nitrogen content estimation models by combining multiple machine learning techniques and stacking integrated learning. The models were built using cotton as the research subject and employed three levels of fusion: feature-level fusion, decision-level fusion, and hybrid fusion. These fusion models were compared to three single data source models. The objective of the study was to enhance the accuracy and stability of nitrogen estimation models, providing theoretical and methodological support for precise monitoring of cotton nitrogen content and efficient fertilizer utilization.

Efficient management of nitrogen during crop growth is crucial in achieving high crop yield and quality (Zhao et al., 2021). Precision agriculture enables the assessment of plant nitrogen supply at the right time and in optimal amounts, ensuring high nitrogen use efficiency while minimizing environmental pollution caused by excessive fertilizer applications. However, there have been limited studies on critical nitrogen concentration in horticultural crops. Chang et al. constructed a critical nitrogen curve for pakchoi to evaluate its growth and nitrogen nutrition status under different soil types and photothermal environmental factors. The results revealed that when the dry matter of pakchoi was below 1.5 t/ha, the critical nitrogen content of the aboveground part was 4.78%, which remained constant. However, when the dry matter exceeded 1.5 t/ha, the critical nitrogen content started to decline. If the yield of pakchoi was below 38.4 t/ha and the plant-critical nitrogen content was below 4.78%, additional nitrogen supply was deemed necessary for pakchoi growth. Additionally, they developed an nitrogen demand model based on the critical nitrogen content, phenotype, and biomass accumulation of pakchoi.

The articles included in this Research Topic have made significant contributions to critical dilution curves and crop nutrition diagnosis. It is important to recognize the crucial role played by plant growth models, such as diagnostic models for predicting fertilizer requirements and critical dilution curves, in the cultivation and production of plants.

## Author contributions

ZL: Writing – original draft, Writing – review & editing. SA-U-K: Supervision, Writing – review & editing. GL: Writing – review & editing.
